# Cross-cultural comparison of perspectives on healthy eating among Chinese and American undergraduate students

**DOI:** 10.1186/s12889-016-3680-y

**Published:** 2016-09-26

**Authors:** Jinan C. Banna, Betsy Gilliland, Margaret Keefe, Dongping Zheng

**Affiliations:** 1 Department of Human Nutrition, Food and Animal Sciences, College of Tropical Agriculture and Human Resources, Agricultural Sciences 216, University of Hawaii at Manoa, 1955 East-West Road, Honolulu, HI 96822 USA; 2 Department of Second Language Studies, College of Languages, Linguistics and Literature, University of Hawaii at Manoa, Moore Hall 570, Honolulu, HI 96822 USA; 3 Shanghai United Family Hospital, 1139 Xianxia Road, Changning District Shanghai, 200336 China

**Keywords:** Young adult, China, United States, Cross-cultural comparison, Food habits

## Abstract

**Background:**

Understanding views about what constitutes a healthy diet in diverse populations may inform design of culturally tailored behavior change interventions. The objective of this study was to describe perspectives on healthy eating among Chinese and American young adults and identify similarities and differences between these groups.

**Methods:**

Chinese (*n* = 55) and American (*n* = 57) undergraduate students in Changsha, Hunan, China and Honolulu, Hawai’i, U.S.A. composed one- to two-paragraph responses to the following prompt: “What does the phrase ‘a healthy diet’ mean to you?” Researchers used content analysis to identify predominant themes using Dedoose (version 5.2.0, SocioCultural Research Consultants, LLC, Los Angeles, CA, 2015). Three researchers independently coded essays and grouped codes with similar content. The team then identified themes and sorted them in discussion. Two researchers then deductively coded the entire data set using eight codes developed from the initial coding and calculated total code counts for each group of participants.

**Results:**

Chinese students mentioned physical outcomes, such as maintaining immunity and digestive health. Timing of eating, with regular meals and greater intake during day than night, was emphasized. American students described balancing among food groups and balancing consumption with exercise, with physical activity considered essential. Students also stated that food components such as sugar, salt and fat should be avoided in large quantities. Similarities included principles such as moderation and fruits and vegetables as nutritious, and differences included foods to be restricted and meal timing. While both groups emphasized specific foods and guiding dietary principles, several distinctions in viewpoints emerged.

**Conclusions:**

The diverse views may reflect food-related messages to which participants are exposed both through the media and educational systems in their respective countries. Future studies may further examine themes that may not typically be addressed in nutrition education programs in diverse populations of young adults. Gaining greater knowledge of the ways in which healthy eating is viewed will allow for development of interventions that are sensitive to the traditional values and predominant views of health in various groups.

**Electronic supplementary material:**

The online version of this article (doi:10.1186/s12889-016-3680-y) contains supplementary material, which is available to authorized users.

## Background

A healthy diet has been well established as a key part of chronic disease prevention and mortality reduction [1–3]. Dietary behavior change is the focus of many health promotion interventions seeking to improve health outcomes, which may motivate individuals to alter their diets by taking actions such as increasing fiber and reducing saturated fat intake [4–6].

In designing health promotion interventions for specific groups, understanding the target population’s health belief systems and views on proper dietary habits is critical, as individuals’ ideals about food have been found to be important determinants of food choice [7]. Studies are needed to examine what diverse populations perceive to constitute a healthy diet [8], with results informing design of culturally tailored behavior change interventions [9]. Previous research examining perspectives on healthy eating in diverse groups has revealed distinct ways in which individuals conceptualize a healthy diet. In a review of the literature examining how qualitative research has advanced understanding of the ways in which people interpret healthy eating, Bisogni et al. identified a number of ideals for healthy eating put forth by diverse cultural groups in multiple countries [10]. Participants in the reviewed studies described healthy eating in terms of specific foods (e.g. fruits and vegetables), food components (e.g. general nutrients, additives), and physical and psychosocial outcomes (e.g. energy, pleasure), among others. Individuals characterize healthy eating in diverse and complex ways, with definitions linking to eating behaviors and spanning various beliefs [11].

While a number of previous studies have examined views on healthy eating using qualitative methods, a review of the literature reveals that only three such studies have focused exclusively on young adults, a group that warrants particular attention with regards to promotion of healthy eating. In the first, examination of undergraduate Canadian females revealed that participants defined healthy eating in accordance with Canadian dietary guidelines and also mentioned organic food, eating with others, and functional foods [12]. The second study, which examined how members of a college men’s ice hockey team experienced the multiple factors influencing their food choices, also revealed participants’ notions of a healthy diet [13]. Most of the players interviewed believed that healthy foods were low-fat foods, and many associated feeling good and having high energy levels with consumption of easy-to-digest foods. Unhealthy foods, in contrast, were described as those such as fries and chips, which may promote feelings of being “bogged down.” Foods such as burgers, pizza, ice cream, cookies and cakes were viewed as tasting good but not healthy [13]. The third study, in Chinese American young adults, indicated that traditional Chinese cuisine was viewed as healthful, conferring benefits such as normal organ function, enhanced immune system, stronger, bones, and longer lifespan [14]. Further research is needed to determine which of these factors are specific to particular cultures.

Understanding perspectives on healthy eating in diverse groups of young adults is of importance in promoting health. During the transition period from adolescence to adulthood (18–31 years), not only is the presence of obesity and unhealthy habits associated with increased chronic disease risk, but young adults also gain independence and establish long-term health behavior patterns [15]. Further, young adults’ current dietary intake is not optimal; a recent study in this population in the U.S. revealed that average daily levels of 6 of the 7 nutrients of concern identified in the *Dietary Guidelines* were lower than recommended [16]. Similarly, in countries undergoing a nutrition transition, such as China, the introduction of a Westernized diet has led to high intake of foods rich in fat and sugar and low in fiber across the lifespan [17–19]. As overweight is a prevalent issue in young adults resulting from such undesirable dietary habits, this population may also engage in dieting practices to achieve weight loss that may potentially have deleterious effects on health [20, 21].

Young adults completing their undergraduate education, in particular, face nutritional issues that warrant the attention of health professionals. The average college freshman has been shown to gain an average of 1.8 ± 0.7 kg in the first year of university attendance [22]. Students who are transitioning from home to the college environment may make poor dietary choices and engage in little physical activity, which may contribute to the weight gain observed [15, 23]. The obesogenic college environment, with availability of all-you-can-eat cafeterias and food high in solid fats and sugars, may play a role in promoting unhealthy choices [22, 24, 25]. Given the particular challenges undergraduate students face in making healthy choices, they represent an important subgroup of young adults to target in promoting sound dietary habits.

To improve dietary habits of young adults, messages about healthy eating have been delivered both through public health guidelines and interventions. In the U.S., the *Dietary Guidelines for Americans* provide evidence-based advice on healthy eating, and outline how people can improve their overall eating patterns [26]. The United States Department of Agriculture (USDA) has also developed MyPlate, a graphic illustrating the 5 key food groups using the familiar image of a place setting for a meal [27]. Other countries have developed similar graphics; for example, the Chinese food guide pagoda conveys the essential components of the diet [28]. As dietary intake of college students has been found to be less than optimal [16], a number of interventions seeking to promote healthy eating have also been conducted [29, 30]. A 2016 systematic review of dietary interventions in university students in diverse world areas, however, noted that out of the twenty studies examined, only one intervention was found to be effective in the long term [30]. In improving upon existing interventions for testing in diverse groups of students, interventions will need to be carefully adapted, evaluated and implemented [31]. Researchers have noted the importance of addressing cultural, social, environmental and psychological forces that influence health behavior in adapting interventions to foster behavior change [32]. Understanding health belief systems is particularly important in tailoring interventions to promote optimal dietary practices, as messages must fit within an individual’s frame of reference to be noticed and processed [33].

As individuals’ eating habits are shaped by the social and cultural contexts of their lives, the ideal diet may be conceptualized differently across diverse populations of young adults. Cross-cultural comparison can reveal valuable findings that may promote cognizance of diverse health belief systems and habits across groups [34]. A comparison of two groups with historically distinct belief systems related to diet and health may be particularly informative in the design of nutrition education messages. Such differences between Chinese and American populations in general, for example, have been well documented. Studies examining the principles underlying the Chinese diet, such as the Yin-Yang belief system of Traditional Chinese Medicine (TCM), reveal stark differences between such principles and those informing the Western diet [35–37]. In a study of three Asian-American groups, for example, all groups expressed the general belief that specific foods have either hot or cold properties and should be eaten strategically to keep one healthy, a concept absent from the Western system [35]. However, the “Westernization” of the Chinese diet has also been well documented, reflecting the influence of global trends [38–43]. Chiva notes that the traditional Chinese meal obeys very strict rules and rituals [44]. However, when economic circumstances allow, people in China eat between meals at any time of day or night, according to supply and demand [44]. The traditional Chinese diet, characterized by high intake of plant-source foods, has been replaced by a diet high in refined carbohydrate, added sugars, fats and animal-source foods [42, 43]. Given the powerful effect of global trends and changing social contexts, it is important to examine to what degree beliefs on healthy diets have converged between countries, or remain distinct. No research to date, however, has considered the intersection of cultural variables with undergraduate students’ views on diet. The purpose of the present study was to describe perspectives on healthy eating among Chinese and American young adults completing their undergraduate studies and identify similarities and differences between the two groups.

## Methods

### Participants and recruitment

Approval was obtained from the Institutional Review Board at the University of Hawaiʻi at Mānoa for an exempt study to allow for analysis of student writing post data collection. Participants were Chinese (*n* = 55) and American (*n* = 57) undergraduate students enrolled in introductory nutrition courses taught by Author 1. The Chinese students were all first- and second-year Food Science majors taking a special two-week introductory nutrition course for which Author 1 was a guest instructor during summer 2014 at Hunan Agricultural University in south-central China, a comprehensive university with approximately 24,000 undergraduate students. Author 1 had been invited to teach the short course to provide the Chinese students with experience taking American-style courses taught in English. This was the first nutrition course the students had taken. The American students were primarily first- through third-years enrolled in Author 1’s general education introductory nutrition course at the University of Hawaiʻi at Mānoa, a comprehensive state university with 14,000 undergraduate students. No compensation was provided for participating in the study.

### Procedures

On the first day of each course, participants composed one- to two-paragraph responses in English to the following prompt: “What does the phrase ‘a healthy diet’ mean to you?” They wrote by hand on paper and were given no additional clarification by the instructor. After 15 min, the instructor collected all students’ written texts. As the task was used as a warm-up activity for the class [45], all enrolled students completed the task. The American students were later invited by Author 2 to participate in the research study and complete the consent form. Fifty-seven out of 121 enrolled students consented to the study. Because the present study was conceived after the end of the course in China, the Chinese students’ essays were approved for use as existing data. A research assistant transcribed the handwritten responses into separate electronic files, maintaining spelling and grammar as written and anonymizing the texts. Author 2 then segmented the transcribed data into 725 distinct T-units (a main clause and related subordinate clauses) and copied the segmented data into an Excel spreadsheet [46]. T-units, “the smallest group of words that can make a move in language” [46], were used as the unit of analysis in order to capture a specific meaning of each piece of text. Larger units such as paragraphs or entire essays contained more than one concept (abstract ideas about eating and nutrition) and therefore could not be labeled clearly with a single code [46]. An example of the segmentation and coding is from Chinese participant 230’s essay is provided in Table 1.

**Table 1:** Example of segmentation and coding from a Chinese participant’s essay

T-unit^a^	Code
First, people should had a balanced diet.	GUIDELINES
Some people think vegetable are not so delicious and don’t eat them.	CONSTRAINTS
However, it has a lot of nutrition and is very good for our health.	BENEFITS
Second, we’d better eat regular meals.	TIMING

^a^Text has been edited for grammar and spelling to enhance clarity

### Data analysis

Directed content analysis using Dedoose qualitative data analysis software (version 5.2.0, SocioCultural Research Consultants, LLC, Los Angeles, CA, 2015) was applied to identify predominant concepts [47]. An initial codebook was developed by Author 1 from concepts that had been noted in previous studies of perceptions of healthy diets [10, 11]. Initial codes included Principles in Dietary Guidelines, Food Properties, Disease Avoidance, Food or Food Groups, Food Components (Nutrients), and Physical Outcomes [10, 11]. Three researchers (Authors 1, 2, and 3) independently coded each essay deductively using the initial codebook [47]. Additional codes were developed inductively when existing codes proved inadequate to describe the data; the researchers iteratively discussed and collaboratively developed operational definitions of new codes. The authors discussed emerging patterns and relationships among codes and then identified higher order categories representing the main messages conveyed in the essays [48].

Authors 1 and 2 then developed a comprehensive codebook to capture the 8 primary categories identified in the open coding (listed in Tables 2 and 3). They independently coded 113 T-units of text (16 distinct essays) for reliability, discussed disagreements, refined the definitions of some codes, recoded, and achieved 81.4 % interrater reliability [46]. Authors 1 and 2 then coded the remaining data set (362 T-units each, with 113 T-units overlapping). All lines of text were coded, with those that did not fall into one of the 8 categories assigned the code “Other.” Lines coded as Other included those that did not relate to the topic of healthy eating at all (such as “I’m really excited for this semester and to learn so much more.”) and lines where the author editorialized or otherwise did not answer the question (such as “Well you should be eating those as a part of a ‘healthy diet.’”). Codes were counted to determine totals for the whole set and for each of the two participant groups, as well as code counts for each essay. Author 4 then reviewed the data to identify lines where Chinese participants drew on traditional Chinese cultural practices.

**Table 2:** Chinese students’ (*n* = 55) descriptions of a healthy diet and exemplifying quotations

Description of a healthy diet (Number of T-units coded/Number of unique essays with this code)	Explanation of code	Exemplifying quotations^a^
Benefits of healthy eating (83/39)	Writer describes positive outcomes related to healthy eating (psychosocial wellbeing, energy, appearance, detox)	“…Eating harmful foods will cause our body to produce harmful substances, so the health foods is very important for a healthy diet, yet the reasonable diet law is very major, such as if we often eat breakfast, we will find our body become very good, but we drop up eat breakfast, we will feel strange, we will fell pain, because our stomach have a lot of gastric acid. The gastric acid will erode our stomach…” “Secondly, you need to eat comfortable, which means eating something you want to eat. A healthy life means happy life, we can access nutrition. From what we like to eat…” “To me a ‘healthy diet’ means consuming foods that make me feel good.”
Guidelines (56/31)	Writer refers to or cites the food pyramid or other official guidelines for healthy eating or concepts from those guidelines; this may include balance, variety, or moderation (either naming a concept or providing an example of the concept)	“A healthy diet is not only the healthy food, but also how much you will eat in a meal. If you eat excessively no matter what food it is, you will be in a bad situation.” “If someone has a healthy diet, he would eat a variety of foods everyday. These foods include grains, meats, vegetables, fruits, eggs, fishes, beans and milk.” “I understand ‘a healthy diet’ is reasonable, healthy food, reasonable nutrition, balanced diet.”
Food groups (50/24)	Writer names specific foods as representative of a food group or food groups, such as fruit, vegetable, meat, dairy, grain, sweets, oils, or plant source foods	“In my eyes, ‘a healthy diet’ means more vegetables and fruits, less meat and oil.” “Swap high fat dairy foods, such milk and cheese for lower fat version.”
Timing of eating (49/21)	Writer names specific meals (breakfast, lunch, dinner) or times when foods should or should not be eaten	“According to many scientists, a person needs more energy and nutrition during the day, and less at night. So a healthy diet may be a large-size breakfast, a medium-size lunch, and a modest-size dinner.” “In China, there is a common saying ‘good breakfast, hearty lunch and litter dinner’ about ‘a healthy diet.’ And many Chinese are following it now. But in my mind, Americans are different. They always have bread and milk in morning. Lunch is coffee or so. And dinner like Chinese lunch, it always more hearty.”

^a^Quotations have been edited for grammar and spelling to enhance clarity

**Table 3:** American students’ (*n* = 57) descriptions of a healthy diet and exemplifying quotations

Description of a healthy diet (Number of T-units coded/Number of unique essays with this code)	Explanation of code	Exemplifying quotations^a^
Guidelines (93/40)	Writer refers to or cites the food pyramid or other official guidelines for healthy eating or concepts from those guidelines; this may include balance, variety, or moderation (either naming a concept or providing an example of the concept)	“A healthy diet to me is one that is balanced. When a girl needs to lose those five extra points they find themselves reading Cosmo magazine looking for a new ‘juice-only’ diet. Although starving oneself might get rid of the weight, it is not healthy nor balanced.” “A healthy diet not only refers to eating well but also takes into consideration of exercising and getting adequate sleep. Those that get more exercise can allow more room to eat more and to then get a variety of food necessary for a healthy lifestyle.”
Non-food practices (58/27)	Writer mentions lifestyle, exercise, sleep as part of healthy diet	“In order to have a healthy way of living it is not just what you put into the body it is what is pushed to get out of it as well. That includes toxins which can be minimized by a combination of exercise and flushing the body using liquids.”
Food components (40/26)	Writer names macronutrients (carbohydrates, proteins, fats), micronutrients (vitamins, minerals), fiber, or water	“Instead of eating too much sugar or fat we might want to choose foods that are “less sugar” or “reduced fat.” “A healthy diet means a consummation of essential nutrients and minerals.” “The best kinds of food may be high in fiber opposed to white flour or apple instead of a cupcake.”
Benefits of healthy eating (33/23)	Writer describes positive outcomes related to healthy eating (psychosocial wellbeing, energy, appearance, detox)	“…Eating only fruits, vegetables, and lean proteins is unrealistic. It is more important to eat that and incorporate those things into everyday eating instead of cutting out ‘bad stuff’ entirely. Becoming obsessive over cutting certain foods out isn’t mentally healthy, so a balanced diet is a healthy diet for both your body and mind.” “To me, healthy means that you are satisfying the physical, mental, and spiritual aspects of health.”

^a^Quotations have been edited for grammar and spelling to enhance clarity

Validity was ensured through 1) Ongoing development of and reference to a codebook containing operational definitions of codes; and 2) Discussion and debate regarding themes identified by the team of three researchers.

## Results

The way in which healthy eating was generally described in Chinese and American participants and exemplifying quotations are provided in Tables 2 and 3, respectively. Findings are reported in the order of prominence in the coded data, with the total number of T-units coded followed by the number of unique student essays noting that code. Table 4 displays the number of students who made statements relevant to each category on healthy eating.

**Table 4:** Number of students who made statements relevant to each category on healthy eating

Description of a healthy diet	US	% total	Chinese	% total
Benefits	23	40.4	39	70.9
Constraints	5	8.8	4	7.3
Drawbacks	8	14.0	14	25.5
Food Components	26	45.6	11	20.0
Food Groups	22	38.6	24	43.6
Guidelines	40	70.2	31	56.4
Non-food practices	27	47.4	15	27.3
Timing	7	12.3	21	38.2

### Perspectives on healthy eating: Chinese participants

#### Benefits of healthy eating

Chinese participants generally described a healthy diet as yielding desirable physical outcomes, such as maintaining immunity and preventing digestive problems. A healthy diet was cited as increasing strength and resistance to ward off illness. In describing the potential consequences of not consuming an optimal diet, chronic conditions such as hypertension, heart disease and overweight were mentioned, as well as problems with the stomach. For example, one participant detailed the negative outcomes resulting from neglecting to eat meals, including gastric acid causing damage to the stomach. Finally, some participants mentioned appearance of the physical body being affected by eating habits, with healthy eating helping one to maintain the right weight and a desirable figure.

Less often mentioned were psychosocial outcomes, such as feeling good and being happy as a result of healthy eating. Eating foods one finds pleasurable was said to be important in living both a happy and healthy life. Participants mentioned that depression may result from suboptimal intake, as there would be a lack of energy to perform activities of daily life and live up to one’s potential.

#### Guidelines

The principles of balance, moderation and variety presented in the Chinese food guide pagoda [28] were highlighted in Chinese students’ essays. To illustrate the concept of a balanced diet, they stated that foods from different food groups should be consumed daily in the correct proportions. Participants also mentioned the need to avoid excessive eating and to consider portion size in planning meals, particularly regarding foods high in sugar and fat. They further noted that different foods should be consumed within each group to maintain varied intake.

#### Food groups

Chinese participants often mentioned fruits and vegetables as important components of the diet. They generally felt that meats should be included in the diet but should be consumed in smaller amounts relative to fruits and vegetables. They further provided specific recommendations with regards to certain groups, such as choosing low-fat dairy and other animal-source foods.

#### Timing

Timing of eating was considered important, with regular meals emphasized. Participants mentioned the value of eating “on time” or “regularly,” and in some cases specified the time of day certain meals should be consumed. For instance, one participant stated that meals were consumed during three set time periods daily: 7 − 8 AM, 12 − 1 PM, and 6 − 7 PM. Not eating on time was reported to negatively affect digestion and absorption and to impede one’s ability to carry out daily activities. Specific foods were said to be more desirable at certain times of day; for example, breakfast may consist of vegetables, fruits, and eggs, lunch of meat, and dinner of fish. The quantity of food at each meal was also viewed to play a role in maintaining health, with larger quantities of food recommended during the day than at night. In some cases, participants stated that breakfast should be the largest meal, followed by lunch, and then dinner.

### Perspectives on healthy eating: American participants

#### Guidelines

American participants most commonly described healthy eating in terms of the principle of balance described in the *Dietary Guidelines for Americans* [26]. Essays often reflected the concept of balancing food groups and consuming meals containing foods from all groups. Participants emphasized the importance of intake of foods from all categories pictured in the food guide, and in many cases perceived elimination of certain groups from the diet for weight loss to be harmful. Participants also discussed balancing consumption with exercise, and addressed the fact that the amount of food consumed should be proportional to energy expended.

#### Non-food practices

Physical activity was often cited as an essential component of a healthy diet. Exercise was said to be important for a number of reasons, which included ridding the body of toxins, expending excess energy, enhancing immunity and maintaining the health of the cardiovascular system. Weight maintenance and loss were also cited as benefits of activity, which many participants said should be performed daily.

#### Food components

Students stated that food components such as sugar, salt and fat should be avoided in large quantities. They mentioned that foods should be rich in nutrients, and often mentioned the importance of vitamins, minerals and protein. Students also distinguished between types of carbohydrates, stating that it is desirable to consume high-fiber foods rather than those high in simple sugars.

#### Benefits of healthy eating

Healthy eating was also said to be important for mental and spiritual health. Participants mentioned that too much focus on the composition of one’s diet may be detrimental to mental health, and that dietary choices should be made for the benefit of both body and mind. A healthy diet, some claimed, helps one “feel good” and wards off depression.

### Cross-cultural comparison of perspectives on healthy eating

In examining the data from each group, similarities and differences were noted. Two common themes recurred in the data: 1) a healthy diet was often described in terms of food groups, with fruits and vegetables commonly mentioned in both groups, and 2) while more predominant in American participants’ essays, principles reflecting the dietary guidelines (presented in the *Dietary Guidelines for Americans* [26] or the Chinese food guide pagoda [28]) were evident in both groups, including balance, moderation and variety. Both Chinese and American participants often mentioned the importance of consuming “more fruits and vegetables,” ideally on a daily basis. These foods were cited as a healthy source of carbohydrate, particularly dietary fiber. In terms of principles in the dietary guidelines, participants in both groups emphasized the importance of balance and moderation. In discussing the importance of moderation, Chinese participants noted that remaining with a sense of hunger at the end of meal may be beneficial. American participants, in contrast, often described moderate consumption as key to incorporating less healthy items, such as foods high in sugar, into the diet in measured amounts. Dietary variety was also important to both groups.

There were also notable differences in participants’ descriptions of healthy eating. While both groups described a healthy diet as requiring restriction, foods to be restricted differed between the two. American participants most often cited carbohydrate-containing foods in their discussion of dietary components that should not be consumed in excess. One participant explained, “When I am trying to eat healthy I avoid all fast food, soda, or sugary drinks, no candy, or unnecessary ‘empty’ carbs.” In several cases, participants distinguished between desirable and undesirable carbohydrate-containing foods. In contrast, Chinese participants were more likely to mention limiting dietary components such as fat and salt. One stated, “Eat smaller amounts of meat, fish and vegetarian alternatives, choosing lower fat options whenever possible.” Meat, specifically beef and pork, was often cited as a food that should be limited.

Compared to Chinese participants’ descriptions of the importance of the timing of eating occasions, American participants’ narratives on this topic reflected some diverse ideas. In general, American participants mentioned timing of eating much less frequently. When mentioned, the concept of “frequent eating” was evident, with three or more meals per day to allow for intake every few hours. This view was reflected in the following explanation from an American student: “Eating small to medium sized portions throughout the day is better for most people’s metabolism than 3 large meals a day and would contribute to maintaining a healthy diet.” Quantity and particular foods to be consumed at eating occasions throughout the day were in most cases not specified, unlike in the Chinese essays, which made reference to appropriate foods for different eating occasions, such as meat at lunch, and emphasized the benefits of hearty portions in the daytime meals and smaller quantities at night.

## Discussion

This study is the first to compare views on healthy eating in two distinct groups of undergraduate students. While both groups described healthy eating in terms of principles guiding current nutrition recommendations and specific foods to emphasize, a number of differences were also identified. The diverse views may reflect food-related messages to which participants are exposed both through the media and educational systems in their respective countries; these findings hold important implications for nutrition education initiatives.

For Chinese participants, timing of eating was considered an important aspect of healthy eating and was mentioned much more frequently compared to American participants. The importance of timing of meals is also emphasized in TCM; classic texts note: “Meals should always be taken at the proper time. This makes them easier to digest. *Yang qi* increases around noon and is weak at sunset. Thus eat a hearty breakfast, a small lunch, and a frugal meal in the evening and night” [49]. Digestive organs are thought to be at their weakest after 6 PM; thus, the evening meal should be small so as not to burden the stomach [50]. Of note, however, recent studies have indicated large shifts in eating patterns in the Chinese population, with the traditional pattern of regularly eating 3 meals per day shifting toward a mixed meals pattern (3 meals plus snacks) [51, 52]. Further, evening has been shown to be a preferred time for snacking [52]. Despite the nutrition transition in China leading to changes in timing of food consumption and content of the diet, findings of the current study reveal persistence of traditional beliefs [18].

Other aspects of Chinese students’ essays also reflected traditional beliefs. For example, physical outcomes were important motivators for consuming a healthy diet, with prevention of digestive problems a common theme. The emphasis on digestive health may also reflect the influence of TCM, which stresses the importance of digestion in maintaining health. In this system, the value of a food is determined both by its nutritional content and the ability of the body to extract that nutrition [53]. Previous qualitative studies conducted in Chinese populations in diverse world areas have revealed the belief that TCM may serve as a guide for health promotion in terms of adoption of a healthy diet [54]. Following these principles, a health professional providing medical nutrition therapy to patients holding Chinese worldviews would consider the natural warm state of the body, especially that of digestion, and would give recommendations that would not incorporate too many foods that are cold either in nature or in physical temperature and therefore may affect digestion [53].

Another possible explanation for the common mention of the importance of a healthy diet for digestive function may relate to the prevalence of functional gastrointestinal disorders in this population. While the degree to which this particular sample had experienced gastrointestinal distress is unknown, a recent study in college students in North China found that functional dyspepsia, irritable bowel syndrome and functional constipation were common [55]. In designing nutrition education interventions for young adults in China, it may be important to draw the connection between diet and digestive function, emphasizing the ways in which dietary intake may influence health of the gastrointestinal tract and prevent conditions such as constipation. Notably, a study that employed qualitative methods to examine beliefs related to obesity prevention revealed similar findings regarding views on physical outcomes of healthy eating among 40 Chinese American young adults [14]. Participants identified enhanced health as the most common benefit of healthy eating, including normal organ function, enhanced immunity, and stronger bones [14]. The Chinese have historically been found to have a strong belief in the relationship between diet and health [56], and stressing the effect of diet on specific aspects of health may be an important component of an intervention. Findings of the current study further reveal traditional beliefs related to diet, suggesting value in promoting traditional practices in Chinese young adults in efforts to prevent chronic disease as China undergoes a nutrition transition.

In addition to the mention of optimal digestive function as a physical outcome resulting from healthy eating, there was also mention, albeit less frequent, of prevention of weight gain and achievement of an attractive figure through a healthy diet. Previous studies have highlighted the strong desire to lose weight sometimes to ultra-thin and unhealthy levels in Chinese college females, despite their being underweight or normal weight in many cases [57, 58]. Exposure in the media to Western beauty ideals has been cited as a factor that may contribute to feelings of fatness and worry about loss of control over eating [59]. Participants’ statements may also in part reflect the public health push in China to reduce rising rates of obesity. Recent economic growth in the country has led to changes in the eating habits of the population and movement toward a more Westernized diet [17–19]. As a result, rates of obesity have increased greatly over the past few decades in most age groups and regions for both men and women [40]. Elucidating the relationship between diet and obesity in nutrition education efforts in China will be an important component of strategies to reverse the current trends. Of note, revised Chinese dietary guidelines have recently been released and have a focus on chronic disease prevention that may assist young adults to make healthy dietary choices [60].

In line with previous findings of qualitative studies focused on defining healthy eating in diverse groups, both Chinese and American students emphasized concepts from the dietary guidelines in their countries, such as balance, variety and moderation. A study of undergraduate Canadian females, for example, demonstrated that participants seemed knowledgeable about the Canadian dietary guidelines, including the four food groups, moderation and variety [12]. Similarly, research in British adults indicated that healthy eating was conceptualized as a balanced and varied diet [61]. Balance and variety were also key concepts highlighted in a study of perceptions of healthy eating in European adults [62]. Such principles have also been cited in defining healthy eating in many other studies [63–67].

With regards to specific concepts from dietary guidelines highlighted in the current study, the principle of moderation was common among both Chinese and American participants’ narratives. Despite this commonality, however, each group had unique perspectives on this topic. The Chinese essays included the idea that a sense of hunger should remain at the end of an eating occasion. American students, in contrast, often cited moderation as a justification for consumption of less healthy dietary components, such as foods high in sugar. Participant statements may reflect somewhat differing views with regards to the meaning of moderate eating among the two groups. Notably, previous studies have highlighted the American focus on quantity in eating and the inclination toward excess [68, 69]. In addition, Chinese immigrants to the United States have been found to have less dietary moderation and higher rates of chronic conditions with acculturation in comparison with less acculturated Chinese counterparts [70, 71]. While both groups noted the importance of limiting intake of foods high in fat and sugar, as well as total calories, the way in which this knowledge is translated into action may differ, with the concept of moderation taking on different meanings. Discussion of appropriate portion sizes and consumption of food within energy needs comprises an important part of nutrition education efforts, in line with the Academy of Nutrition and Dietetics’ “total diet approach” to healthy eating [72].

American participants frequently referenced balance, which is a dominant theme for basic nutrition education in the United States [26]. The current food icon, MyPlate, is based on the concepts of balance, variety and moderation, helping consumers to stay within their daily calorie needs, make smart choices from every food group, and find a balance between food and physical activity [27]. American students’ essays emphasized both the necessity of consuming a balanced diet and of offsetting consumption with sufficient exercise. The importance placed on physical activity is concurrent with previous research. In a qualitative study of 49 undergraduates at a Midwestern university, for example, LaCaille et al. found that students identified many motivators for physical activity, including being in shape, relaxation, improved mood and energy, and improved self-esteem [73]. While it is evident that the present group of young adults had knowledge with respect to the importance of exercise and current recommendations, considerable effort is still required to encourage implementation of these guidelines. As indicated in previous studies, many young adults in the U.S. do not adhere to the *Dietary Guidelines* and are not meeting the recommendations for physical activity [16, 74]. As knowledge is only one factor among many that impact behavior, it may be important to focus on other factors in promoting physical activity in young adults in the U.S., such as psychological processes (perceptions, values, attitudes) that may influence health-related choices, and environmental supports that may foster desirable behavior [29, 73, 75].

In addition, American participants’ views on which foods to restrict also reflect current trends in the nutrition field. In recent years, a number of diet plans involving limiting carbohydrates have gained popularity, including the Atkins Diet [76], the Paleo Diet [77], and the South Beach Diet [78]. In participants’ narratives, a number of carbohydrate-containing foods were identified as targets for restriction. Of note, some participants mentioned restricting carbohydrates generally, without discussion of particular carbohydrate containing foods that would be particularly important to limit, such as sugar-sweetened beverages. In nutrition education initiatives targeting young adults in the U.S., it may important to debunk myths about dietary components such as carbohydrates, emphasizing the science behind current recommendations. Indeed, a previous study seeking to identify the needs of U.S. college students for development of nutrition education programs, identified the concept of “nutrition myths and facts,” both generally as well as on specific topics in the field, among topics of importance [79].

Several themes not typically addressed in nutrition education interventions also emerged in both groups, such as those relating healthy eating to psychosocial outcomes. Participants mentioned the importance of a sound diet in maintaining mental health, feeling good, and warding off depression. In their review of the literature on interpretations of healthy eating, Bisogni et al. also identified meanings related to psychosocial outcomes, reflecting the complexity of beliefs on healthy eating and connection to various facets of life [10]. Based on the emergence of these themes, the researchers acknowledged the importance of addressing factors beyond the scope of typical nutrition education initiatives [10]. To reflect the results of previous studies, mental health has been addressed in a number of nutrition programs implemented in recent years, including the MOVE! weight management program for veterans [80] and the COPE [Creating Opportunities for Personal Empowerment] cognitive behavioral skills building TEEN [Thinking, Emotions, Exercise, and Nutrition] program designed for adolescents [81]. Dietary change has been cited as having potential to improve mental wellbeing in undergraduate students, an important consideration for young adults [82].

Findings revealing the differences and similarities in views of healthy eating in Chinese and American young adults hold implications not only for health professionals working with these populations in their home countries, but also for professionals addressing the needs of diverse populations. Dietitians in the U.S., for example, often work to address the needs of immigrant populations that are less acculturated in terms of adapting host country dietary customs, and must therefore understand the predominant views of health in the immigrants’ country of origin. The Asian population grew faster than any other race group in the U.S. between 2000 and 2010, with Chinese making up the largest component of this group [83]. Given this, as well as the increase in obesity and related chronic conditions in the Chinese American community, researchers have highlighted the immediate need among health professionals to understand Chinese culture, with its specific beliefs, attitudes, and behaviors, as it relates to obesity risk [14, 84]. Gaining this understanding will be particularly important not only in meeting the needs of Chinese American young adults, but also in serving Chinese international students in U.S. colleges and universities, a fast-growing population representing the majority of international students in the U.S [85]. Given the particular nutrition-related challenges that college students face, the current study provides important information that may inform nutrition education interventions in a diverse at-risk group.

This study has several limitations. First, only 2 distinct groups of young adults representing 2 world areas were included. To further compare and contrast views on healthy eating in young adults, groups in other regions such as Europe, Africa or South America could be included. Secondly, participants included in the study were drawn from only one university in each country, and had self-selected into an introductory course on nutrition, limiting the generalizability of the results. The sample was, however, heterogeneous in terms of age and gender, and did allow for the discovery of important information regarding the diverse views of healthy eating in 2 distinct populations. Finally, the methods employed did not allow for examination of student responses in terms of whether students demonstrated complex thinking that linked multiple relevant concepts. Future studies may identify issues of complex thinking to provide greater depth of analysis.

## Conclusions

In working with Chinese and American young adults, nutrition educators will need to take into account the distinct motivators for healthy eating identified in participants’ descriptions of a healthy diet. For example, in the Chinese population, long-term physical outcomes such as enhanced immune function may be important to address, and concepts of TCM may be integrated into educational programs. For an American audience, the benefits of consuming carbohydrate-containing foods in fueling the body may be a useful area of focus. The current study also revealed the potential variability in the way in which the 2 populations interpret nutrition-related concepts such as moderation, and the need for further research to gain an in-depth understanding of meanings of such terms and how individuals put them into practice. Future studies may also further examine themes that may not typically be addressed in nutrition education programs in diverse populations of young adults and incorporate findings into nutrition education program development. Gaining greater knowledge of the ways in which healthy eating is viewed will allow for development of interventions that are sensitive to the traditional values and predominant views of health in various groups and incorporate the tastes and perspectives of the targeted population.

## Additional file

Additional file 1: American (1−57) and Chinese (200−254) Participants’ Essays. All essays for participants in China and the US. (DOCX 160 kb)

## References

[1] Ford ES, Bergmann MM, Boeing H, Li C, Capewell S (2012). Healthy lifestyle behaviors and all-cause mortality among adults in the United States. Prev Med.

[2] Capewell S, O'Flaherty M (2011). Rapid mortality falls after risk-factor changes in populations. Lancet.

[3] Beaglehole R, Bonita R, Horton R, Adams C, Alleyne G, Asaria P, Baugh V, Bekedam H, Billo N, Casswell S (2011). Priority actions for the non-communicable disease crisis. Lancet.

[4] Tuomilehto J, Lindstrom J, Eriksson JG, Valle TT, Hamalainen H, Ilanne-Parikka P, Keinanen-Kiukaanniemi S, Laakso M, Louheranta A, Rastas M (2001). Prevention of type 2 diabetes mellitus by changes in lifestyle among subjects with impaired glucose tolerance. N Engl J Med.

[5] Kroenke CH, Caan BJ, Stefanick ML, Anderson G, Brzyski R, Johnson KC, LeBlanc E, Lee C, La Croix AZ, Park HL (2012). Effects of a dietary intervention and weight change on vasomotor symptoms in the Women's Health Initiative. Menopause.

[6] Hebert JR, Hurley TG, Harmon BE, Heiney S, Hebert CJ, Steck SE (2012). A diet, physical activity, and stress reduction intervention in men with rising prostate-specific antigen after treatment for prostate cancer. Cancer Epidemiol.

[7] Furst T, Connors M, Bisogni CA, Sobal J, Falk LW (1996). Food choice: a conceptual model of the process. Appetite.

[8] Paquette MC (2005). Perceptions of healthy eating: state of knowledge and research gaps. Can J Public Health.

[9] Barrera M, Castro FG, Strycker LA, Toobert DJ (2013). Cultural adaptations of behavioral health interventions: a progress report. J Consult Clin Psychol.

[10] Bisogni CA, Jastran M, Seligson M, Thompson A (2012). How people interpret healthy eating: contributions of qualitative research. J Nutr Educ Behav.

[11] Falk LW, Sobal J, Bisogni CA, Connors M, Devine CM (2001). Managing healthy eating: definitions, classifications, and strategies. Health Educ Behav.

[12] Su J, Levy-Milne R, House J (2006). Definitions of healthy eating among university students. Can J Diet Pract Res.

[13] Smart LR, Bisogni CA (2001). Personal food systems of male college hockey players. Appetite.

[14] Liou D, Bauer KD (2007). Exploratory investigation of obesity risk and prevention in Chinese Americans. J Nutr Educ Behav.

[15] Nelson MC, Story M, Larson NI, Neumark-Sztainer D, Lytle LA (2008). Emerging adulthood and college-aged youth: an overlooked age for weight-related behavior change. Obesity.

[16] McDaniel JC, Belury MA (2012). Are young adults following the dietary guidelines for Americans?. Nurse Pract.

[17] Astrup A, Dyerberg J, Selleck M, Stender S (2008). Nutrition transition and its relationship to the development of obesity and related chronic diseases. Obes Rev.

[18] Zhai F, Wang H, Du S, He Y, Wang Z, Ge K, Popkin BM (2007). Lifespan nutrition and changing socio-economic conditions in China. Asia Pac J Clin Nutr.

[19] Li Y, Zhai F, Yang X, Schouten EG, Hu X, He Y, Luan D, Ma G (2007). Determinants of childhood overweight and obesity in China. Br J Nutr.

[20] Neumark-Sztainer D, Wall M, Larson NI, Eisenberg ME, Loth K (2011). Dieting and disordered eating behaviors from adolescence to young adulthood: findings from a 10-year longitudinal study. J Am Diet Assoc.

[21] Neumark-Sztainer D, Wall M, Eisenberg ME, Story M, Hannan PJ (2006). Overweight status and weight control behaviors in adolescents: longitudinal and secular trends from 1999 to 2004. Prev Med.

[22] Vella-Zarb RA, Elgar FJ (2009). The ‘freshman 5’: a meta-analysis of weight gain in the freshman year of college. J Am Coll Health.

[23] Greene GW, White AA, Hoerr SL, Lohse B, Schembre SM, Riebe D, Patterson J, Kattelmann KK, Shoff S, Horacek T (2012). Impact of an online healthful eating and physical activity program for college students. Am J Health Promot.

[24] Greaney ML, Less FD, White AA, Dayton SF, Riebe D, Blissmer B, Shoff S, Walsh JR, Greene GW (2009). College students’ barriers and enablers for healthful weight management: a qualitative study. J Nutr Educ Behav.

[25] Nelson MC, Story M (2009). Food environments in university dorms: 20,000 calories per dorm room and counting. Am J Prev Med.

[26] US Department of Health and Human Services. Dietary Guidelines for Americans. 2015.

[27] United States Department of Agriculture. ChooseMyPlate. 2015. http://www.choosemyplate.gov/. Accessed 15 Feb 2016.

[28] Chinese Nutrition Society. The Chinese Dietary Guidelines. 2007.

[29] Kelly NR, Mazzeo SE, Bean MK (2013). Systematic review of dietary interventions with college students: directions for future research and practice. J Nutr Educ Behav.

[30] Deliens T, Van Crombruggen R, Verbruggen S, De Bourdeaudhuij I, Deforche B, Clarys P (2016). Dietary interventions among university students: A systematic review. Appetite.

[31] Bhopal RS (2006). The public health agenda and minority ethnic health: a reflection on priorities. J R Soc Med.

[32] Resnicow K, Baranowski T, Ahluwalia JS, Braithwaite RL (1999). Cultural sensitivity in public health: defined and demystified. Ethn Dis.

[33] Green LW, Kreuter MW (2005). Health Program Planning. An Educational and Ecological Approach.

[34] Torsch VL, Ma GX (2000). Cross-cultural comparison of health perceptions, concerns, and coping strategies among Asian and Pacific Islander American elders. Qual Health Res.

[35] Harrison GG, Kagawa-Singer M, Foerster SB, Lee H, Pham Kim L, Nguyen TU, Fernandez-Ami A, Quinn V, Bal DG (2005). Seizing the moment: California's opportunity to prevent nutrition-related health disparities in low-income Asian American population. Cancer.

[36] Manderson L (1987). Hot-cold food and medical theories: overview and introduction. Soc Sci Med.

[37] Lee MM, Shen JM (2008). Dietary patterns using Traditional Chinese Medicine principles in epidemiological studies. Asia Pac J Clin Nutr.

[38] Gunewardene A, Huon GF, Zheng R (2001). Exposure to westernization and dieting: a cross-cultural study. Int J Eat Disord.

[39] Woo KS, Chook P, Raitakari OT, McQuillan B, Feng JZ, Celermajer DS (1999). Westernization of Chinese adults and increased subclinical atherosclerosis. Arterioscler Thromb Vasc Biol.

[40] Xi B, Liang Y, He T, Reilly KH, Hu Y, Wang Q, Yan Y, Mi J (2012). Secular trends in the prevalence of general and abdominal obesity among Chinese adults, 1993-2009. Obes Rev.

[41] Wang H, Zhai F (2013). Programme and policy options for preventing obesity in China. Obes Rev.

[42] Popkin BM, Adair LS, Ng SW (2012). Global nutrition transition and the pandemic of obesity in developing countries. Nutr Rev.

[43] Popkin BM (2009). Global changes in diet and activity patterns as drivers of the nutrition transition. Nestle Nutr Workshop Ser Pediatr Program.

[44] Chiva M (1997). Cultural aspects of meals and meal frequency. Br J Nutr.

[45] Bean J (2001). Engaging Ideas: The Professor’s Guide to Integrating Writing, Critical Thinking, and Active Learning in the Classroom.

[46] Geisler C (2004). Analyzing streams of language: Twelve steps to the systematic coding of text, talk, and other verbal data.

[47] Hsieh HF, Shannon SE (2005). Three approaches to qualitative content analysis. Qual Health Res.

[48] Merriam SB (2009). Qualitative Research: A Guide to Design and Implementation.

[49] Jing HDN (1986). The Yellow Emperor’s Classic of Medicine.

[50] Kastner J (2004). Chinese Nutrition Therapy : Dietetics in Traditional Chinese Medicine (TCM).

[51] Wang Z, Zhai F, Du S, Popkin B (2008). Dynamic shifts in Chinese eating behaviors. Asia Pac J Clin Nutr.

[52] Wang Z, Zhai F, Zhang B, Popkin BM (2012). Trends in Chinese snacking behaviors and patterns and the social-demographic role between 1991 and 2009. Asia Pac J Clin Nutr.

[53] Blair J, Meredith M, Plotnikoff GA (2012). Introduction to traditional Asian therapeutic diets: two enduring perspectives. Minn Med.

[54] Chung VC, Ma PH, Lau CH, Wong SY, Yeoh EK, Griffiths SM (2014). Views on traditional Chinese medicine amongst Chinese population: a systematic review of qualitative and quantitative studies. Health Expect.

[55] Dong YY, Chen FX, Yu YB, Du C, Qi QQ, Liu H, Li YQ (2013). A school-based study with Rome III criteria on the prevalence of functional gastrointestinal disorders in Chinese college and university students. PLoS ONE.

[56] Kaptchuk TJ, Tomalin S (2000). The Web That Has No Weaver: Understanding Chinese Medicine.

[57] Madanat HN, Hawks SR, Campbell T, Fowler C, Hawks JL (2010). Young urban women and the nutrition transition in China: a familiar pattern emerges. Glob Health Promot.

[58] Tao ZL (2010). Epidemiological risk factor study concerning abnormal attitudes toward eating and adverse dieting behaviours among 12- to 25-years-old Chinese students. Eur Eat Disord Rev.

[59] Peat CM, Von Holle A, Watson H, Huang L, Thornton LM, Zhang B, Du S, Kleiman SC, Bulik CM (2015). The association between internet and television access and disordered eating in a Chinese sample. Int J Eat Disord.

[60] Chinese Nutrition Society. The Chinese Dietary Guidelines. 2016.

[61] Povey R, Conner M, Sparks P, James R, Shepherd R (1998). Interpretations of healthy and unhealthy eating, and implications for dietary change. Health Educ Res.

[62] Margetts BM, Martinez JA, Saba A, Holm L, Kearney M, Moles A (1997). Definitions of 'healthy’ eating: a pan-EU survey of consumer attitudes to food, nutrition and health. Eur J Clin Nutr.

[63] Lupton D, Chapman S (1995). “A healthy lifestyle might be the death of you”: Discourses on diet, cholesterol control and heart disease in the press and among the lay public. Sociol Health Illness.

[64] Health Canada, Sage Research Corporation. Qualitative Investigation of Canadians’ Understanding of and Attitudes Towards Nutrition and Healthy Eating. 2003

[65] Kean A, Willetts A (1996). Concepts of Healthy Eating: An Anthropological Investigation in South East London.

[66] Bouwman LI, te Molder H, Koelen MM, van Woerkum CM (2009). I eat healthfully but I am not a freak. Consumers' everyday life perspective on healthful eating. Appetite.

[67] Falk LW, Bisogni CA, Sobal J (1996). Food choice processes of older adults. J Nutr Educ.

[68] Rozin P (2005). The meaning of food in our lives: a cross-cultural perspective on eating and well-being. J Nutr Educ Behav.

[69] Rozin P (2000). Why we’re so fat, and French are not. Psychol Today.

[70] Liu A, Berhane Z, Tseng M (2010). Improved dietary variety and adequacy but lower dietary moderation with acculturation in Chinese women in the United States. J Am Diet Assoc.

[71] Tseng M, Wright DJ, Fang CY (2015). Acculturation and dietary change among Chinese immigrant women in the United States. J Immigr Minor Health.

[72] Freeland-Graves JH, Nitzke S (2013). Position of the academy of nutrition and dietetics: total diet approach to healthy eating. J Acad Nutr Diet.

[73] Lacaille LJ, Dauner KN, Krambeer RJ, Pedersen J (2011). Psychosocial and environmental determinants of eating behaviors, physical activity, and weight change among college students: a qualitative analysis. J Am Coll Health.

[74] Centers for Disease Control and Prevention. CDC - Facts - Data - Physical Activity - DNPAO. https://www.cdc.gov/physicalactivity/data/facts.htm. Accessed 15 Feb 2016.

[75] Shepherd R, Towler G (2007). Nutrition knowledge, attitudes and fat intake: application of the theory of reasoned action. J Hum Nutr Diet.

[76] Atkins. How the Atkins Diet Works. https://www.atkins.com/how-it-works. Accessed 15 Feb 2016.

[77] The Paleo Diet. The Paleo Diet - Live Well, Live Longer. http://thepaleodiet.com/. Accessed 15 Feb 2016.

[78] Agatston A (2005). The South Beach Diet.

[79] Cousineau TM, Goldstein M, Franko DL (2004). A collaborative approach to nutrition education for college students. J Am Coll Health.

[80] Kinsinger LS, Jones KR, Kahwati L, Harvey R, Burdick M, Zele V, Yevich SJ (2009). Design and dissemination of the MOVE! Weight-Management Program for Veterans. Prev Chronic Dis.

[81] Hoying J, Melnyk BM, Arcoleo K (2016). Effects of the COPE Cognitive Behavioral Skills Building TEEN Program on the Healthy Lifestyle Behaviors and Mental Health of Appalachian Early Adolescents. J Pediatr Health Care.

[82] Richards, AP S. Diet and wellbeing in undergraduate students. Appetite. 2014;83(362):362.

[83] US Census. The Asian Population: 2010 Census Briefs. 2012. https://www.census.gov/prod/cen2010/briefs/c2010br-11.pdf. Accessed 15 Feb 2016

[84] King GL, McNeely MJ, Thorpe LE, Mau ML, Ko J, Liu LL, Sun A, Hsu WC, Chow EA (2012). Understanding and addressing unique needs of diabetes in Asian Americans, native Hawaiians, and Pacific Islanders. Diabetes Care.

[85] Yan K, Berliner DC (2010). Chinese international students in the United States: demographic trends, motivations, acculturation features and adjustment challenges. Asia Pac Educ Rev.

