# Supplementary material for: Cross-cultural comparison of perspectives on healthy eating among Chinese and American undergraduate students
Source: BMC Public Health. 2016 Sep 26;16:1015. doi: 10.1186/s12889-016-3680-y (PMC5037860; doi:10.1186/s12889-016-3680-y)
Supplement: Additional file 1: — American (1−57) and Chinese (200−254) Participants’ Essays. All essays for participants in China and the US. (DOCX 160 kb) [file 12889_2016_3680_MOESM1_ESM.docx]

**American (1-57) and Chinese (200-254) Participants’ Essays**

001

A healthy diet to me means balancing out all different food categories from the food pyramid; eating an equal amount of protein and carbs and lessening sweets and fatty foods.

Typically while on a healthy diet, it is important to not overeat one type of food such as just eating red meat and no chicken or fish.

A healthy diet also includes eating at least 3 meals a day and not skipping any (breakfast, lunch, or dinner).

002

“A healthy diet” mean to eat healthyly,

eat foods that are less calories and fats because the cause of overweight or diseases are relate to the high calories foods.

We should eat wisely,

take a look at the nutrition info before eating the foods because a “healthy diet” is start off from the foods that we choose to eat.

Instead of eating too much sugar or fat we might want to choose foods that are “less sugar” or “reduced fat”,

it will be a ‘healthy diet’ to me.

003

A healthy diet, to me, means that you are eating a meal (or meals) that have the proper food groups represented every day.

This could mean that you are eating proteins, fruits, vegetables, starches, dairy, etc.

This also means that you don’t overeat foods that are harmful to your body, such as sugars, calories, fat, and other processed goods.

I also think a big part of a healthy diet would be getting enough sleep, drinking lots of water, and exercising regularly.

All of those components are pivotal to maintaining a healthy lifestyle in my opinion.

004

A healthy diet is a nutrition plan that benefits the body.

Healthy diets can contain many things from different food-groups.

Personally I feel a healthy diet should contain fruits, vegetables, wheats, meat, and a healthy source of hydration.

Although many people think about just food when they hear the phrase healthy diet, I believe exercise is also a huge part in supplementing a healthy diet.

005

I think that a healthy diet means balancing your food intake by eating the right amounts for you.

A healthy diet consists of all the healthy foods needed

but I think it includes all the other types of foods also.

Whether it be “junk” food or may something healthy like an orange.

I think both types are needed for both being healthy and increasing the quality of life.

I think that a healthy diet has a good balance of all the major category groups of food,

as in, only eating protein isn’t necessarily a good diet, especially if you are missing out on all your other food groups.

A healthy diet not only refers to eating well but also takes into consideration of exercising and getting adequate sleep.

Those that get more exercise can allow more room to eat more and to then get a variety of food necessary for a healthy lifestyle.

006

A healthy diets means to me eating the right amount of fruits and vegetable in a day.

It is also eating “portioned” sized foods and drinking plenty of water.

Some protein is also good.

Snacking should also be very limited and sugary drinks as well.

Today it seems that a healthy diet is nearly impossible with all the ads and the consumerism economy we live in.

But it is essential to our daily lives to try and make it a habit to eat healthy.

007

To me, a healthy diet is the same as a healthy lifestyle;

eat a well-balanced meal as well as exercise regularly.

Eating healthy means getting the right amount of vegetables, fruits, and protein in your system.

This makes people feel good

and if you feel good you look good and perform well.

I think that a little sugar and cheat days are fine as long as you don’t overeat.

Exercising regularly is the other part to a healthy diet.

30 minutes a day is all you need to stay healthy.

008

To me “a healthy diet” is a diet that is balanced

and you body gets the nutrition it needs to stay healthy.

I think you can eat bad foods on a healthy diet once in a while, but not every single day.

A healthy diet consists of eating fruits, vegetables, dairy, whole grains, and protein of just the right amount.

Eating bad can make your body feel all weak.

However, eating a healthy diet can make your body feel great.

009

A healthy diet to me is one that is balanced .

When a girl needs to lose those five extra points they find themselves reading cosmo magazine looking for a new “juice-only” diet.

Although starving oneself might get rid of the weight, it is not healthy nor balanced.

Eating the right amount of fruits, veggies, carbs, calories, proteins, and fats are what make a healthy diet.

Also recognizing that one might need a few more iron-filled foods than others can help when eating healthy

010

Healthy diet to me means eat right and exercise your body.

It getting good food and nutrition

so your body can function at it highest level.

Healthy diet is also mixing things up

so I can’t just have veg you need meat also.

It just not going over board in one thing,

but spreading the love and working out.

011

A healthy diet consists of you making right choices about what you eat.

The whole idea of not eating does not consist of a healthy diet

what is important is that you keep control of your food intake

and when you do have something unhealthy to eat, then you can always work it off afterwards.

Nutrition and exercise go hand in hand w/ consuming a healthy diet.

Keep the important nutrients in the body and take away the unnecessary foods by exercising regularly but not to the point of overdoing it.

012

A healthy diet consists of a good amount of vegetables, fruits, carbohydrates and wheats.

I think that intaking enough calories is an important part of a healthy diet.

To me, having a healthy diet does not just consists of healthy eating habits, but also exercise.

Intaking the essential nutrition and vitamins are also important.

Having a good mindset/healthy eating habits are essential for maintaining a healthy diet.

013

To me, “a healthy diet” means many things.

First let’s analyze the word “healthy”.

To me, healthy means that you are satisfying the physical, mental, and spiritual aspects of health.

You are eating (consuming) enough micro + macro nutrients to offset your caloric output.

You exercise enough to maintain a strong immune + cardiovascular system.

BMI plays into physical health.

Hopefully you are engaging your brain enough to keep it active and sharp.

School is good for that.

You need enough sleep to rest your body + mind in preparation for the day to come.

Then we must look at “diet.”

This is the part where I must personally disagree with the terminology being used.

Often times, “diet” involves making unreasonable, sometimes extreme lifestyle changes in order to achieve a healthy body weight.

The abrupt changes almost never stick once you stop “dieting.

To me,”a healthy diet” is not something should try to have.

Instead, I feel people should try to achieve a healthy lifestyle.

014

Firstly, a healthy diet means a diet that contains all of the required nutrients (or most of them).

It should incorporate carbohydrates, protein, minerals, vitamins, and even fat in an appropriate amount.

In addition, these nutrients have to be in balance in their composition in the diet.

Secondly, ‘a healthy diet’ is a diet that you eat on a REGULAR basis.

To elaborate, you have to eat a regular amount of food, regularly on time.

That way, your body is able to absorb the “healthy” nutrients appropriately, at the time it needs them.

015

To live a healthy diet means to have a healthy lifestyle.

To be having a diet of good health requires more than eating good food.

It’s more so about how your stomach and body feels from your lifestyle.

Sure you may eat meats everyday,

but, are you carefully watching what you eat?

For example, the regular caloric intake a day, or making sure if you have gained the right amount of nutritional needs; carbs, protein, etc..

Also, a diet is not healthy if you don’t exercise along with it.

016

A healthy diet means that I’m meeting my caloric, fat, carbohydrate, +protein goals.

It consists of fruits, vegetables, good source of complex carbs (is brown rice, etc.), healthy fats (avocado), +protein.

It also means that I’m aware of what the nutritional macros are in whatever I consume

and therefore, I am able to decide whether I want to eat that food or not.

This also applies to what I drink, as water is the majority of what I consume.

017

A healthy diet in my opinion means “eating mindfully’.

I believe you should eat until your body feels full + happy, and also by eating the right things, that fuel your body with energy.

Even though eating right is an important thing, I believe it’s ok to eat what you want, but within boundaries.

Also, I think a healthy diet consists of eating things that will make you more alert and energized.

Besides eating healthy, I think it is important to have a regular amount of exercise to release endorphins that give you energy as well.

Overall, I think having a healthy diet means to eat mindfully, with awareness of what you are putting into your body.

018

To me a “healthy diet” means consuming foods that make me feel good and are loaded with nutrients.

Growing up involved in sports helped me maintain habits of healthy eating since early age.

As I grew up I was diagnosed with celiac’s disease and can no longer eat any gluten or wheat.

This was a big struggle for me to completely cut all gluten out of my diet since most of the best food is made with bread!

I have now been gluten free for 2 years and feel so much better!

Now my diet consists of all veggies, lean meats, fruits, nuts, beans, ect.

I have come to the point where eating junk food makes me sick and lathargic.

Becoming gluten free really helped me become interested in food science and how what we eat can affect us in so many ways!

I’m really excited for this semester and to learn so much more :)

019

A healthy diet means eating the right foods and having the right amount of food intake per day.

That means eating the recommended amount of each food group.

It also means staying active to keep your body in shape as well as running properly throughout the day.

Being able to stay away from unnecessary foods such as candy + soda will help to keep the calories away that is not needed in ones diet.

020

I believe a “healthy diet” means getting your daily nutrient intake through fruits, vegetables, and other non-processed foods.

I did a little bit of research and learned that pre-packaged foods are preserved foods aren’t good for your body.

So a healthy diet includes only a little bit of this.

Being in a healthy diet also means being able to indulge in the “not-so-happy” items such as ice cream and sweets/desserts.

Having a healthy diet means being disciplined in what you put in your body.

It means keeping your body happy.

021

A healthy diet means to me that you feel good almost the entire day.

You can do sports or activities of what ever you want and still feel well.

In general I want to know what I am eating and know how it affects my body.

Meats, dairy, fruits and vegetables I assume you are part of a healthy diet.

Just feeling good and not sleepy and grogry is what I think is a healthy diet.

022

A healthy diet is a well balanced intake of the nutrition necessary to keep you body functioning.

it assist in preventing illness and injury and keeps all bodily systems active and in a decent condition.

Healthy diets do not need to be reserved for weight loss and should be a part of everyday living.

It should be accompanied by other smart life choices such as regular exercise and the absence of drugs.

This diet is the one best suited for an individual's specific lifestyle and should be tailored to their body’s needs.

023

A healthy diet means alot to me.

Whatever we consume as human beings constitutes how well our bodies responds to activity.

A healthy diet means a consummation of essential nutrients and minerals.

Our bodies need to sustain our moving bodies and our more-or-less “active” lives.

The famous “moderation is key” phrase limits and regulates unnecessary food intake.

Giving ourselves too much or too less is detrimental to our physical and mental health.

024

A healthy diet means a long life, full of happiness, opportunity and adventure.

Food and nutrition is the basis of our existence.

If we do not nourish our bodies or feed it what it needs we will be miserable our entire lives - fighting illnesses, feeling depressed with no energy to take on life's daily challenges, and worst of all, not able to reach our full potential.

I personally have had to overcome many health problems during my short life.

I know first-hand how very important it is to eat healthy and to maintain a healthy lifestyle.

I’ve been positively influenced my whole life by my sister, who is in medical school for naturopathic medicine.

I am on a very healthy diet - yes, because I have to be, but also because I WANT to be.

I want to have a long, fulfilling life without problems hindering me like they already have.

I am excited to learn more throughout this course

and I hope to improve myself with each passing day.

025

A healthy diet, for me, is a really important part of life.

This includes having a well balanced meal with fruit, vegetables, grain (whole grain), and even a small portion of trans fat occasionally.

Being healthy is not eating as little as possible,

it’s eating the best kinds of food as possible.

The best kinds of food may be high in fiber opposed to white flour or apple instead of a cupcake.

A healthy diet means a healthy lifestyle.

026

The phrase “healthy diet” means incorporating foods into ones daily lifestyle that benefit the person by being nutritious.

There should be a serving of carbs that matches the physical activity of the individual, along with a low sugar intake of natural sugars.

A “healthy diet” also includes eating at proper times of the day and not snacking in the middle of the night, or starving one’s self.

Eating small to medium sized portions throughout the day is better for most peoples metabolism than three large meals a day and would contribute to maintaining a healthy diet.

Lastly, having a healthy diet does not only mean to uphold these standards and focus on natural foods, but also to embrace a lifestyle that follows suit with the food choices.

027

The phrase ‘a healthy diet’ means to me, is something your body is comfortable with.

You are consuming all the right nutrients your body needs as a part of your everyday life.

Eating a healthy diet doesn’t necessarily mean you can’t eat something that isn’t “organic” or “green”.

Will you should be eating those as a part of a “healthy diet”.

But eating by portions, staying away from greasy fast food and what not is the opposite of an healthy diet.

By what I mean about your body comfortable with, it is well-nourished and able to let you function everyday.

By using the healthy food as a energy source to keep you up

028

The phrase ‘a healthy diet’ to me does NOT means being the thinnest, the skinniest, the curviest, the leanest, or even the most muscular.

I believe that a healthy diet is consuming the average/right amount of calories your body needs, and also having a balanced diet w/ the expected amount of meats, poultry, dairy, starches, and vegetables consumed on a daily basis.

In addition to having a balanced diet and balanced calories, exercise, sleep and water are also factors to living a healthy life.

With the right amounts of all these things, I believe a person would be qualified as “healthy”

029

In my opinion, a healthy diet is comprised of several important factors.

The first, and most important factor is a diet comprised of non-processed foods.

In short, if you read the ingredients label and have no idea how to pronounce the word, it is probably best not to eat it.

Another important factor is to eat frequently.

Smaller meals or snacks every few hours, 200 calories within the first two hours of being awake, and protein within twenty minutes of working out.

A third important factor is to drink lots of water throughout the drug, and even more with working out/ intensity of workout.

In a nutshell, these are some important basic nutrition guidelines.

These are some more detailed facts to be taken into considerations such as male vs female (amounts of fats), height-weight-metabolism (calories), type of exercise/ goals and any pre-existing conditions such as low blood sugar, allergies, etc.

030

To me a healthy diet is eating the proper nutrients that your body needs to function at the best of it’s ability.

It means eating proper serving portions and at proper times of the day.

Having a healthy diet to me is alliminating as much artificial and processed foods from your diet as possible.

A healthy diet is consisted of REAL foods like lean protein, complex carbs, veggies, fruits, grains, and lots of water.

Having a healthy diet affects the way you feel everyday.

Having a healthy diet is eating clean but also being aware of what you are eating, and what you need to consume to be your healthiest version of yourself.

031

A healthy diet is to have meals on regular schedule (like 3 meals a day) and also make sure the person takes varieties of food such as veggies + meat + rice/bread.

In addition to that, avoid junk food such as McDonalds as much as possible.

Also, never let the desire brings your mind away.

For instance, have too much desserts on one day or the preference on oily food is prohibited.

The most important thing for “a healthy diet” is to be clever on whatever that’s going into your mouth.

032

A healthy diet means consuming an adequate amount of the nutrients our bodies need in order to sustain energy levels.

This greatly varies depending on our physical activity and other contributing factors (disease, genes, etc)

I am a vegetarian which means that I must pay close attention to ensure that I receive enough protein and healthy fats in my diet.

Avoiding excessive amount of sugar, fat, and complex carbohydrates is also important in achieving a healthy diet.

033

A healthy diet is a life style and not just a phase.

It takes time and research to actually put nutritious foods into the human body.

A healthy lifestyle is a combination of many food groups in order to give the body what it needs to perform daily functions.

In order to have a healthy way of living it is not just what you put into the body it is what is pushed to get out of it as well.

That includes toxins which can be minimized by a combination of exercise and flushing the body using liquids.

034

To me, a healthy diet (to put simply) consists of whole foods that are free of chemicals and preservatives.

A healthy meets (but doesn’t exceed) the recommendations for CHO, protein, and lipids.

If this diet is low of crap (Carbonated, Refined, Artificial, and Processed), then I would consider it to be healthy.

Ultimately, a healthy diet comes from the earth it’s natural state.

Get your carbs from veggies, fruit, and grains.

Get your protein from lentils and fish (or poultry).

Lastly, get your healthy fats from nuts & fish as well.

035

‘A healthy diet’ to me consists of a variety of foods.

For years it was usually the same foods over and over

but recently I decided to change that and mix up what I eat.

Specifically, a healthy diet of mine is having at least every other meal have some colored food that isn’t golden brown on my plate.

I have never been a person to actively eat fruits and vegetables so adding some to every other meal is a rather large step.

My hope is by the end of the semester the amount of those types of foods consumed will increase.

036

My body knows best.

Our species has spent thousands (understatement) of years developing feedback systems that tell me to eat, sleep, and drink.

That being said, not all the foods or beverages that we have at our disposal now were available 30,000 years ago.

So long as food, with variety and moderation, is organic/natural than life will go on smoothly.

037

To me, a healthy diet means that you are taking in enough calories for your body type, and getting enough nutrients.

You incorporate food from all of the food groups and eat them in moderation.

I also believe that you should balance what you eat with physical activity.

Being active will help to make sure you burn off what you take in.

038

In my opinion, a healthy diet is more than just a means to lose weight or gain weight (what ever your situation may be).

I see it as, mainly, a lifestyle and a way to properly give your body the nutrients it needs to function properly.

You could easily be what people perceive as healthy, based on outward appearance, but be unhealthy internally because you eat junk and just don't gain weight.

So mainly, a healthy diet to me is the proper nourishment of your body by eating properly to get the nutrients your body needs to be healthy internally as well.

039

In my opinion, a healthy diet goes beyond what you eat.

I believe that to have a healthy diet, you must also abstain from large amounts of toxins that are in drugs and alcohol.

Typically, a healthy diet should consist of eating well-balanced meals containing all different food groups.

Not eating junk food should be mandatory when satisfying a healthy diet.

I also believe that to have a healthy body you must also have a healthy mind,

and avoiding stress is a big component to having a healthy mind along with meditation practice and exercise.

These are all things that everyone can do so

therefor I believe it’s possible for everyone to have a healthy diet.

040

To me, a ‘healthy diet’ means well-rounded, nutritious diet.

It means healthy foods that good for your body.

A healthy diet should consist of foods from all the major food groups and contain all the vitamins your body needs to thrive.

A healthy diet should not include excessive sugar or carbs, but should maintain a balance of everything.

Having a healthy diet is very important to keep your body well and functional.

041

The phrase “healthy diet” means that you are getting all of the needed nutrients and vitamins to nourish your body.

I think that having a healthy diet allows you to have enough energy throughout the day to complete your daily tasks.

I feel like having a healthy diet is not necessarily a “diet” but a lifestyle where you are fueling your body appropriately.

Also where you can get most of your nutrients and vitamins through plants rather than pill form.

042

I believe that a healthy diet consists of a balance of the different food groups (as shown in the food pyramid).

Thus, we should have the least amount of sugars and a high amount of grains with all other kinds of food in between.

A diet is not just food however,

liquids such as, and maybe most importantly we need water to help bodily function.

To pay respect to individuals, many people also require special diets therefore to them,

and to everyone else a healthy diet can also be defined as enough of the daily essential nutrients needed for proper bodily function (proteins, water, minerals, nutrients, etc.)

043

A healthy diet means various things to many people.

I believe that a healthy diet refers to a lifestyle choice.

A diet tends to follow the latest fad among people.

Learning how to have healthy habits is much more ideal than a fad diet.

Learning basics such as proteins, carbs, and lipids is essential for a person to know in order to know how to take care of themselves.

Sometimes eating healthy can be pricey

but there are ways people can learn to work around those challenges.

When someone decides to discipline themselves to have healthy eating habits, they are benefiting and adding longevity to their lives.

044

A “healthy diet” to me means that one is able to monitor what they eat and also know what the food does to their body.

It also means that one has a regular exercise routine.

Healthy eating combined with the knowledge of eating correctly is what I think a healthy diet is.

I think when someone works out regularly and eats healthy they will continue to have a healthy diet throughout life.

045

To me, a healthy diet means a regular consumption of food & beverages that help one's body grow, repair, and maintain.

A healthy diets details vary from person to person, as certain individuals may need more of something then another individual.

For example, a petite pregnant woman would have a different necessary diet than an obese man trying to lose weight.

So, a healthy diet boils down to being whatever your body needs more of to get where you're going!

046

To me, a ‘healthy diet’ means a balanced diet.

Eating foods from all of the different food groups in appropriate proportions.

Also, eating healthy includes looking out for what is in your food and how it is produced.

Today, a lot of the food we in take has extra chemicals that we don’t need.

Therefore, a healthy diet shouldn’t just be a bunch of fruits and vegetables, but quality ones that don’t have GMOs.

047

To me, a healthy diet means being balanced.

Eating what’s good for you is important,

but eating only fruits, vegetables, and lean proteins is unrealistic.

It is more important to eat that and incorporate those things into everyday eating instead of cutting out “bad stuff” entirely.

Becoming obsessive over cutting certain foods out isn’t mentally healthy,

so a balanced diet is a healthy diet for both your body and mind.

048

When I think of the phrase a healthy diet, I think of 3 key concepts.

A healthy diet to me consists of a willingness to evaluate an individual’s diet, the ability to research what is good and food to put in your diet and perseverance to apply a diet to an individual’s new lifestyle.

A willingness to evaluate our individual’s diet is the first thing I think of in correlation to a healthy diet.

Diets can be good or bad

and the first step for a person is to see which diet they have.

It is much easier to eat unhealthy because there are more resources.

However, it takes a person effort to make their diet healthy.

Next, it is hard to maintain a healthy diet especially in college with limited funds & resources

it takes perseverance and a background in nutrition to keep a healthy diet & maintain a healthy lifestyle.

049

A healthy diet consists of a balance of all types of foods.

Not a lot of sugary stuff like soda and candy.

More fruits and vegetables.

Not too much salty foods.

It also means having daily exercise and making healthy choices, watching what you eat and how much is consumed.

050

To me, maintaining a healthy diet means that you are consuming as much nutrients, fats, protein etc. as your body needs to survive & thrive.

I believe everybody is different

& therefore the amounts of sugars, fats, protein etc needed to keep them healthy may vary.

A healthy diet consists of finding a balance between fruit & vegetables, carbs & dairy, sugar & fats, all to overall benefit you in the best possible way.

051

To me, a healthy diet means that we eat our daily amount of vegetables, fruits, proteins, carbs, basically whatever is on the food pyramid.

We should make sure that it is a balanced diet and not eat too much of one things.

I do not count protein powders and such supplements as part of a healthy diet.

I don’t believe people really need those supplements unless it is in a vitamin form, for example Flinstone vitamins or calcium chews.

A healthy diet just means getting the necessary calories and vitamins without over eating or excluding a food group.

052

In my perspective, I believe a healthy diet means eating clean, proportioning your food and limiting yourself from other foods that can negatively affect the body.

A healthy diet is for the benefit of your body,

it is consuming the right amount of food while performing good exercises in between.

Eating right, intaking nutrients, vitamins, consuming more vegetables and fruits compared to carbs.

When I hear “healthy diet” I think of the word “balance” and “dicipline” for the good of your own body.

This is what a healthy diet means to me.

053

To me, being skinny doesn’t always mean you have a healthy diet.

A healthy diet is being able to eat a nutritious meal and feeling very energized and satisfied once you gain the required nutrients needed.

It’s always remembering to eat your fruits and vegetables, yet still have some sweets.

I feel that when you have a healthy diet, you know what is in your food.

Not only knowing the ingredients, but knowing what you’re getting from it.

054

It means sustaining/maintaining your healthy habits that helped you live this long.

It doesn’t only apply to eating and making smart eating choices, but also healthy life decisions that are food for you.

If something in life isn’t making you happy then that’s not a healthy diet you are living.

Making a committment to short-term challenges for long-term goals.

055

To me a healthy diet means two things, portion control and balanced nutrients.

Portion control means not eating more food than your body needs, or can handler in a single sitting.

Allow your body time to digest is important.

Also balancing nutrients such as carbohydrates & fats, as well as making sure you eat the proper servings of fruits and vegetables, meats, dairy, and grain each day and at each meal.

056

A healthy diet is comprised by many factors.

one factor of a healthy diet would be having sufficient amount of intake within all major food groups.

The food groups consist of fruits/veggies, protein, wheat, daily and fats.

Also it is very important that you handler your food with moderation.

This is important because overeating or undereating can drastically effect your health.

Also working out at least 3 times a week is important because keeping your body healthy contributes to a healthy diet.

These are just a few things in a healthy diet.

057

The phrase healthy diet can be misleading.

People view it in different ways and some people have food alergies.

To me, a healthy diet consists of 3 or 4 meals per day drinking a lot of water and avoiding processed foods.

Your meals should consist of a source of protine and vitamins.

When I am trying to eat healthy I avoid all fast food, soda, or sugary drinks, no candy, or unnecessary “empty” carbs.

Organic fruits and veggies as well as poultry products make up most of a healthy diet.

200

A healthy diet, in my point of view, is a kind of balance diet.

To keep healthy, people should not eat too much nor too little in one meal, should not eat only vegs or meat instead of take both of them.

A healthy diet requires people to eat what they need but not what they want.

Eating at the same time in every day is considered healthy

for the disordered eating timetable will hurt the stomach.

Individual will feel better and better through a healthy diet, physically as well as psychologically.

Because a healthy diet could help to keep fit and stay away from many disease such like stomachache etc.

201

I think “a healthy diet” means diet balance,

it is also mostly every meal has fruits, vegetables, and meat.

And average daily intake of food.

I think eating habits can help the human body the digestion and absorption everyday.

Secondly, oil for a meal. Play an importance significance because how a lot of nupure oil to appear on the market.

202

As for me, I think ‘a healthy diet’ mean you should eat something what good for you.

Everyone need a healthy life, and have a healthy diet is primary of it.

So, it is necessary to eat some healthy foods.

Even junk food and other high energe food taste good, most people prefer them.

They are bad for our health.

And there are wide range of diseases are responsible for them.

Therefore, we should eat more vegetable and fruits to keep health.

Besides, we need to do more exercise and stay mental health.

203

In my life, ‘a healthy die’ means eat some healthy food And has a good healthy habit.

Like breakfast, I think vegetables, fruits, and eggs.

I think it makes us ability.

About lunch, we should take some meat and eat more than breakfast and dinner.

Finally, about dinner, we should eat some fish.

Of course, we all know, food is not enough.

So we also must take exercise.

204

Healthy Diet

In my opinion, a healthy diet mean two point.

Firstly, you must eat healthy.

As we all know, the nutrition that our body need is different.

So, we must understand how reasonable mix diet.

Try the best to let u eat healthy.

Secondly, you need eat comfortable, which means eat something you want to eat.

A healthy life mean happy life,

we can access nutrition. From what we like to eat.

It means you can healthy diet with a happy feeding.

205

A Healthy Diet

Actually, when I was a child, in my eyes, a healthy diet means have meats, vegetables and a soup in a meal.

When I gradually grew up, the definition of healthy diet has become different.

As far as I am concerned, healthy diet need to make protein, fat, vitamins and cellulose etc.

It is equally important that the volume of each nutrients intake must keep in certain range.

Keeping good obsorb of those nutrients, people should promise certain proportion of those.

206

In my eyes, “a healthy diet” means eating regularly and robust.

That’s to say the human not only should eating on time, but must without junk food, like ice cream, barbecue.

Nowaday, a corte number of girls who want to make them more charming chose on diet to lose weight.

But I think it’s harmful because nothing is more important than out health.

For the junk food. Most of teenagers think it’s delicious,

however, their health problem increasing by.

All in all, I think “a health diet” is important for our life.

Because because our body is what you eat.

207

“a healthy diet” is meaning that we reasonable absorb nutrition everyday.

a healthy diet can regulate physical defect.

Prevent disease some extence maintain and supply energy. by three meals a day.

Provide nutrient by healthy food.

Nowadays, a number of people like the fast food.

I think those are bad food; like snack is delicious food,

but snack is bad to healthy in the main.

208

I think a healthy diet means a lot to us.

Firstly, it is no doubt that a healthy diet could make you a healthy body.

Love pencil to work or study.

Then, a healthy diet bring you a good mood.

Have a smile face to anyone.

209

In my opinion, ‘a healthy diet’ mean use scientific methods to absorb nutrition.

‘a healthy diet’ can keep balance on human body system by regularly exercise and healthy eating habit, which avoid absorb oo much fat and serious disease.,

because human body can only absorb limited nutrition.

So that over take food doesn’t means healthy.

210

I think the healthy diet is very important.

If we don’t keep a healthy diet. There will be something bad to us. Such as fall ill. Been fat and so on.

So the healthy diet is very important.

It can make us keep health. Strengthen the resistance power. And keep a favorable body.

211

Nowadays, a healthy diet is significant to most of us.

People are about what they chose to eat and whether it is healthy or not.

For me, ‘a healthy diet’ means what kind of food I am going to eat and can they provide me enough energy, proein, and vitamin.

A healthy diet is not only the healthy food, but also how many you will ate in a meal.

If you ate excessively no matter what food it is, you will be in a bad situation.

212

When it is come to the topic healthy diet.

You know, as the development of economy in China. Healthy diet has aroused progressively.

People concern there re so many phrases. Which originate from thousands of yours China ancestor. Illustrate the spirit that they continuously explore for the unknown.

Ha. Not finished…

213

“A healthy diet” take an important role in our life.

We need eat on time for our each meals, then, develop this habit.

Can make us personal laws, better, healthy foods can give us more power to store more difficult things, and makes you look confident all day.

The most important is healthy diet can keep us a healthy body.

214

Speak of the healthy diet, usually would associate to nutrient balance.

This is significant.

After all, balance of nutrients in the body keep normal physiological activities.

But, this is most essential condition of healthy diet.

Intake source must correspond to food hygiene standardization.

Most important is taste good.

In my crew, perons don’t like unsavory food.

215

It’s a big question to me.

In China, the healthy food is mean not very delicious.

So the delicious food may be not very healthy, like the fried food and the vegetable.

So, the healthy diet is very important to me.

the vegetable and low-fat food which should I eat.

We must to keep my health

and it makes me more happy.

That’s all. Thank you.

216

In my opinion, a healthy diet is the key reason leading us to a healthy life.

A healthy diet makes me grow stronger.

It also good for our body.

Keep eating fruits and vegetables everyday is an example.

217

Healthy is important to every one,

so we should concern about healthy diet.

Healthy diet can help us to prevent diseases.

We should eat more fruits and vegetables everyday,

and we do also have to eat more nutrition foods to keep our health.

218

What is the meaning of healthy diet?

A large of qwise ideas are appeared.

In my opinion, the healthy diet is too eat on the basis of regular pattern and take in balanced nutritional such as eating third one day, vegetable and a little meat.

We cannot eat a little for losing weight, or we will have the opposite consequence.

However, plenty of public fails to realize this problem.

It is not a benifit phenomenon.

So we should study hard and try to change this situation.

219

Nowdays, people hold that a balant diet can bring us a good lifestyle eating.

What tips for healthy eating?

There are two impants we will tell to you.

Firstly, you should eat a variety of foods.

It is significant to choose foods from all of food groups and a variety of different foods.

Besides, you also should try new foods or new ways of preparing foods.

Secondly, don’t forget to eat more whole grain product.

For example, cereals, whole wheat breads.

As we all now, grain products give your body things which it needs to stay healthy like vitamins, energy.

220

A heathy diet means that the food must be healthy and the nutrition could be absorbed in our body.

The food of a healthy diet is including carbohydrate, protein, fat and so on.

Everyone gains energy through these food, because they need working through consuming energy.

Nowadays, more and more people becoming unhealthy, because of the unhealthy diet.

Such as hypertension, overweight, stomach illness and so on.

If we don’t pay attention to our diet, our health would be in danger.

In a word, a healthy diet is important for us.

221

In my opinion, I believe “a healthy Diet” is a balanced eating habit in our daily life.

I mean when people take in nutrition, they should consider the appropriate size that they could get.

According to many scientists, person needs more energy and nutrition during the day, and less at night.

So a healthy Diet may be a large-size breakfast, a midum-size lunch, and a modest-size dinner.

Why does people be advised to eat like this,

perhaps there are so many reasons.

One is that you keep fit if you do this.

Other is that you can also keep a good body in life.

You will have a perfect appearance and strong system to prevent any ills.

222

“A healthy diet” means that different aged people get a good match between meat and fruit.

This is my first ideal course, rare elements are not unnecessary, like fluorine.

Something that cannot get fluorine food.

Secondly, I think “a healthy diet” means regular eating habit.

Thirdly, less eating, everyday we only need get the energy that our body need is enough, because much energy metaboliz in our body means much metabolized trash damage to our body.

223

Firstly, eating regularly.

For example, you should eat breakfast in 6 to 8am.

Don’t eating too much junk food although it is look like delicious.

Many people like me usually want to eat junk food.

I think you can eat it but just few times.

Then, you should concentrate on when to eat, where to eat for instance.

In nothing, you should eat many foods like meals, rather than meats.

In lunch, you can eat more meats.

Drinking enough water everyday.

Exercising everyday.

Early to sleep, early to get up.

224

As to me, “a healthy diet” that means to having healthy food and have a good dietary habit.

In our daily life, almost of people don’t attach importance to have a healthy diet.

As a example some people usually taking junk food rather than having food in school mess.

And some people just a lazybone,

they don’t having breakfast, after 12:00 am, they taking lunch in diracty.

I think keep in health is importance to everybody,

we just keep exercise and eating some healthy food like vegetables fruits and so on.

Only in this way, can we have a healthy body to meet the life.

225

In china, there is a common saying “good breakfast, hearty lunch and litter dinner” about “a healthy diet.”

And many chinese had following it now.

But in my mind, American is defference.

They always have bread and milk in morning.

Lunch is coffee or so.

And dinner like chinese lunch, it always more hearty.

What’s more, a “a healthy diet” should

226

To my opinion, the balance of eating is the phrase “a healthy duet” mean to me.

As we all know, human might get kinds of food every day,

public menu included vegetables, meat, egg, milk,

so, how to keep people’s eating equilibrum are important.

During the research, scientist find the best project for people’s daily meal are 3 meats, 70% vegetable and eggs at that program,

and neither man or weman can get the nutrition for them body and keep them health.

227

A healthy diet mean to me that is a balanced diet and enough exercise.

You know, only the nutrition eating you have, the strong body you will get.

So, the pork for us is not good for us.

We need more varity of kinds, like beef, fish, shutton and chicken.

The more varity of kinds meat you have, the more strong body you will have.

But the meat is not the only food we have to eat.

We also need many vegetables, as it anmis fiber which can help us easier to get the prion of the meat.

Also the vegetables have large of v.c.

it can help us break older and help we have shinne face.

The many fruits also have this benefit like apples, bananas and cheese.

The other thing I believe need to have is enough exercise.

That is bad for us if we only chose eating and refused training.

The necessary sport can make our body more pretty,

and withing the energy used, we do not worry about we will get a fat weight, which is a good way to choose lose weight.

That is all I support the means about a healthy diet.

228

A healthy diet is different for different people, like “vegetarian”

in their opinion, people should not eating meats, eggs, and other food from animals,

but Human is a kind of omnivorous animal,

there are some amino acid cannot get from vegetables and fruits but they are important for human life.

So “Healthy diet” means sutiable, balanced and public common.

And there is another nord “food medical,”

maybe human can use eating some speacill food (from nature) to protect body away from illness.

229

Health is a very important thing to people.

A healthy diet is a good way to keep fit.

As the diet tower is shown, people must eat a lot of grains each day.

Also, people must eat plenty of vegetables, fruit and some meat, fish, dairy product very little fat and sugar every day.

You can eat as much as you can in breakfast or lunch,

but you should stop eating before you feel full at dinner time,

or else your stomach can not absorb all the food.

And you will have problems with your stomach.

A good diet can make you healthy and away from illness.

230

“a healthy diet”

In my eyes, “a healthy diet” means more vegetables and fruits, less meat and oil.

However, people always have bad lifestule and diet.

Such as, breakfast and lunch eating together;

people like eat meat and just eat less vegetable, sometime they not eat fruits so how to have a healthy diet is a problem.

Relecent method should be carried out to this problem before things go worse.

First, people should had a balanced diet.

Some people think vegetable are not so delicious that not eating it.

However, it have a lot of nutrition and it is very good for our healthy.

Second, we’d better eat regular meals.

231

A healthy diet means a healthy lifestyle for me.

Because, a good dietary habit benefit to our body.

Besides, it can help us to lose weight.

If people have unhealthy food, such as junk food.

High fat food may lead to obesity or having a heart problem, even cancer

this is why the reason of the important of a health diet.

232

A healthy diet is common phase in our daily life.

In my vision, how eating and what eating are important for this word.

Eating the food of nutrition is health for us when eating it is also ritual to keep health.

For example, eating eggs and drinking milk every day.

The lunch can inject lot of foods appropriately.

To eat little in every night.

This health diet will keep healthy body and increase the resistance that preventing the illness.

233

“A healthy diet” mean to me is some foods eaten by me which including vegetable and meat and cereal.

Because vegetable and cereal is rich in DF (dietary fiber), which can make human’s body become more healthy.

And meat can provide protein to human’s body

of course, all food as above also supply energy for our body.

In addition, I think “A healthy diet” need we eat food regularly.

234

In my opinion, the phrase “a healthy diet” means eating the right foods and eatting regularly.

If someone has a healthy diet, he would eat a variety of foods everyday.

These foods include grains, meats, vegetables, fruits, eggs, fishes, beans and milk.

Suitable intake of protein, fat and carbohydrate are benefit to our body.

Another assential is to drink an adequate amount of water each day to replace the natural loss of body fluids.

To sum up, this is the mean of a health diet by me.

235

From my point of view, “a healthy diet” is very important for us.

Balancing everyday basies is the root factor to our health.

Enjoy a variety of different foods from each food group and eat regularly.

Including breakfast, the fruit and vegetables should make up one third of the food you eat.

Unrefined starchy (e.g. bread, rice and potatoes) should also make up about a third of your food intake.

Eat smaller amounts of meat, fish and vegetarian alternatives, choosing lower fat options whenever possible.

Swap high fat dairy foods, such milk and cheese. For lower fat version.

Limit food in the fatty and sugary group, and use sparingly.

236

As far as I’m concerned, a healthy diet is very important for our health.

Firstly, we should have a balanced diet like eating vegetables, even if they are not delicious, but also have a lot of nutrition.

Secondly, eat regular is better for us.

Regular meals throughout the day give us enough energy to carry out our daily activities. Thirdly, don’t eat junk food.

Because junk food is unhealthy to us.

In a word we should eat the right kinds of food and do not eat any of the unhealthy food.

237

How to have a healthy diet.

Harty healthy diet is good for our healthy.

It can make your body strong and healthy.

But how to have a healthy diet?

In my opinion, first of all, we should the diet at the right time.

Chaotic mealtimes will affect our body’s normal digestion and absorption.

It’s bad for your health.

Second having a healthy diet means eating healthy food, such as all somes of grain green vegetable and fruit.

Different food has different nutrition which our body need.

Finally we should not eat to much or too little for every meal.

Overeat or dieting is very harmful for our stomach and health.

238

Health is the guarantee of one’s happiness.

Without a healthy body, on cannot do what he want to, not to mention accomplishing his goals in life.

One can keep fit in three ways.

First he is to pay attention to hygiene,

stay away from viruses, which means one should wash hands, take baths frequently, eat and drink properly,

stay away from bad life habits, such as smoking and overworking, which severely damage the body organs.

Secondly, one is to exercise refularly.

He may go jogging, swimming, bicycling, playing basketball and so on.

Exercises will improve one’s ability to resist diseases.

And last but not least he should be optimistic hearing no difficulties and obstacles and always being in high spirits.

Only in good healthy as well as in high spirits can we great the future which is full of competition and challenges and strive to achieve our ambitions energetically.

239

As we know, any people need food to live.

Because it can provide energy to support us to act many things, just like play basketball, swimming, study, and so on.

So a healthy diet are so important which let’s us more stronger.

As for me, the healthy diet means safely food.

Not only include safely food for us but also belongs to delicious

we know healthy diet can efficiently to control the weight of your body.

Far away many commons ills.

240

I think a healthy habit will be regular.

A regular bedtime is extremely important.

Than, a healthy diet is more important.

As a study of food science students we should have a certain understanding for the choice of food.

As a result, the status of all kinds of food for our life will be understood clearly,

summer has come,

I think yogure that is refreshing and healthy drinks is our first choice

and a wide variety of yogure became our difficult choices.

In my opinion, the frozen and solid-state yogure is the highest nutritional value.

The same, you still need a regular rising time.

241

Vegetable and fruit are important for a healthy diet.

If you want to have a good health, that dietary habit also important,

you should understanding the food groups and becoming more aware of calories and the roles that specific nutrients play in a healthy diet.

In my daily life I try to eat more vegetable, eating huge amounts of wheat, and eat fruit after a meal,

of course, you also should increase your exercise, to keep you body health.

Some people think that different, because the health food always not delicious

so it you can find delicious healthy food it couldn’t be better.

242

Personally, a healthy diet is eating regularly and exercise often.

In common situation I divide meal time into three stages, 7a.m. to 8a.m., 12a.m to 1p.m., and 6p.m to 7p.m.

However, I might become hungry before next stage coming.

Facing such phenomenon I have to eat some traits, which can provide energy to me and avoid intake too much because when the next eating time comes I cant eat any more.

In my opinion, exercises are necessary for my daily life,

it not only build my body, but also keep my health and my figure.

243

In my opinion, a healthy diet reflects all aspects of our lifes.

For example, some people often do exercises are more healthy than others, because it can be favorable for their digestive system.

In addition, people seldom eat junk food like fried food and barbecue which is a way of a healthy diet.

In other words, to realise a healthy diet, we should experience a long-term insist in resisting many temptations in our lifes.

244

As is well-known the diet is linked with the human health.

Of you inattention your eating habits, you would become ill-health.

So we should hare a healthy diet to stay healthy.

As far as I’m concerned, a healthy diet means that we should eat more fruits, vegetables and drink more water, but not to eat junk food such as barbecue, fry and so on.

In order to have a good body, we must pay attention to diet.

245

For most people what they care most probably is the taste and the convenience.

And they are less care in the terms of nutrition, health and hygiene.

Some plausible approach is often a misunderstanding, who let you always believe eating proverbs, many of them are full of one-sideness lies.

Sometimes a good diet can take the place of drugs and may have a better effect, although it need a relatively long time.

But today, more and more research results show that the damage to the human health most of disease are caused by improper diet

so “a health diet” needs to be taken attention by people.

246

I understand “a healthy diet” is reasonable, health food, reasonable nutrition, balanced diet.

Neither too much food, nutrition, but also to ensure that the energy for the human body normal physiological metabolish.

Healthy eating also includes food hygiene refused to junk food, strict control of harmful chemicals in the residues in food.

247

I believe the healthy diet must have healthy foods and reasonable diet law,

for example, we eat harmful foods will cause our body produce harmful substances,

so the health foods is very important for health diet,

yet the reasonable diet law is very major, such as if we often eat breakfast, will will find our body become very good,

but we drop up eat breakfast, we will feel strange,

we will fell pain, because our stomach have a lot of gastric acid.

The gastric acid will erode our stomach,

so, we should eat health foods and have reasonable diet law.

248

In my opinion, “a healthy diet” is your healthy eating habits.

Firstly, I just drink a cup of milk and bread in breakfast.

It have protein, glucose and other which can provide enough energy.

In lunch and dinner, I may eat varicous food.

It can provide most of things which are important for human living like vitamin, waters and other.

For me, “a healthy diet” is a healthy lifestyle.

It can guide what we should eat, how to eat well every day.

249

With the development of society, the public began to concentrate on the healthy diet,

so, it’s also important for me.

In my opinion, the healthy diet is the basic of vital movement,

it will ensure we can achieve the abundant nutrient content.

If you want to own the healthy body, I think the healthy diet should be important.

For me, the healthy diet not only a hobby, but also the manner of living.

Only we have the healthy diet, we can live better and more disciplinary.

By the way, I think healthy diet is to say the reasonable diet

and the vegetable and fruit are the main parts.

250

The healthy diet mean nutrition is balanced

when our diet of everyday get better and more nuitient, this good life-style guarantee we have a healthy body.

Since the nutriology was born, the persons life-style have become to more regular.

In order to longerity, people are seem to attention to our own diet style,

so, the healthy diet is mean to human nutrition and health.

251

Nowadays, the healthy diet is more and more important in our daily live.

I think the most crucial aim of healthy diet is the food which we intake could sustain our basic action and would not cause any painful to our body.

That must contain the balance of these six nutrients.

Moreover, a healthy diet must has ability to improve our beneficial system in our body like immune system, emergency system and so on.

That means we could intake some functionality foods to help us get a more healthy life.

For example, a large number of tomatoes could help us resist the heart disease

and the ferment foods could enhance our digestive system.

In sum, a healthy diet must good for our health.

252

I believe healthy diet can give me a healthy and strong body.

Health is most important for man, regularly and healthy diet can help me have a healthy body.

Healthy diet tell me cant eat too much or less,

we must have a adequeate intake.

Healthy diet is part of human nutrition,

so I think I need hard to learn this science, and learn more about healthy diet to help me and our friends or family have a good diet habits

I also believe healthy diet can extend our life span.

253

The concept of “healthy diets” consist of a lot of aspects.

I think the most important is taking food on time everyday.

Then you will get necessary energy to finish essential life activities,

of course, nowadays, the increasingly number of people advocate that we should have more meals a day but less food at each.

Because some survies showed that the superfluous food we take will translate into toxin in our body.

Another point of “a healthy diet” I believe that we should notice a balanced nutrition

we all know carbohudrate, lipid, protein, and so on.

They are the most important and basic compound.

We are indispensable to learn and remember the chinese food guide pyramid.

For example, we should take more frumomtum vegetables and fruits. Instead of salts and lipid.

By the way “remain a sense of hunger” is normal.

Last but not least doing some decent exercise is also a part of “healthy diets.”

It can help our body expelling of toxin and consume the spare energy.

Then it also can enhance our immunity.

Therefore, having a healthy diet equate to have a healthy body.

254

To eat much vegatable.

To do more exersices everyday.

To eat less beff and poak

The choclata is not healthy

The fruits is great

Less oil and salt.
